# Dermal Mesenchymal Stem Cells (DMSCs) Inhibit Skin-Homing CD8+ T Cell Activity, a Determining Factor of Vitiligo Patients’ Autologous Melanocytes Transplantation Efficiency

**DOI:** 10.1371/journal.pone.0060254

**Published:** 2013-04-05

**Authors:** Miao-ni Zhou, Zhi-qing Zhang, Ji-long Wu, Fu-quan Lin, Li-fang Fu, Sui-quan Wang, Cui-ping Guan, Hong-lin Wang, Aie Xu

**Affiliations:** 1 Department of Dermatology, The Third People’s Hospital of Hangzhou, Hangzhou, P. R. China; 2 Neuroscience Institute, Soochow University, Suzhou, Jiangsu, P. R. China; 3 Shanghai Institute of Immunology, Institute of Medical Sciences, Shanghai Jiao Tong University School of Medicine, Shanghai, P. R. China; Ajou University, Republic of Korea

## Abstract

We here investigated the efficiency of autologous melanocyte transplantation of 23 vitiligo patients by focusing on perilesional skin homing CD8+ T lymphocytes, and studied the potential effect of dermal mesenchymal stem cells (DMSCs) on CD8+ T cell activities *in vitro*. Out of 23 patients with the autologous melanocyte transplantation, 12 patients (52.17%) had an excellent re-pigmentation, 6 patients (26.09%) had a good re-pigmentation, 5 patients (21.74%) had a fair or poor re-pigmentation. CD8+ T cells infiltrating was observed in the perilesional vitiligo area of all patients. Importantly, the efficiency of the transplantation was closely associated with skin-homing CD8+ T cell activities. The patients with high number of perilesional CD8+ T cells or high level of cytokines/chemokines were associated with poor re-pigmentation efficiency. For *in-vitro* experiments, we successfully isolated and characterized human DMSCs and skin-homing CD8+ T cells. We established DMSCs and CD8+ T cell co-culture system, where DMSCs possessed significant inhibitory effects against skin homing CD8+ T lymphocytes. DMSCs inhibited CD8+ T cells proliferation, induced them apoptosis and regulated their cytokines/chemokines production. Our results suggest that vitiligo patients’ autologous melanocytes transplantation efficiency might be predicted by perilesional skin-homing CD8+ T cell activities, and DMSCs might be used as auxiliary agent to improve transplantation efficacy.

## Introduction

Vitiligo is an acquired skin de-pigmentation disorder characterized by the progressive development of skin areas without functional melanocytes. Although the precise etiology of vitiligo is still not fully addressed, auto-immune destruction, oxidative stress and genetic factors have been suggested [1]. Reactive oxygen species (ROS) play important roles in Vitiligo pathology [2]. Reduced catalase enzyme levels and increased hydrogen peroxide levels were seen in vitiligo perilesional skin [3], [4], [5]. ROS were shown to impair the activity of tyrosinase and the corresponding repair mechanisms [2], ROS can also affect autoimmune activity [2]. Meanwhile, sustained ROS are able to induce melanocytes apoptosis [6]. Meanwhile, the autoimmune pathogenesis has been proposed as one of the main causes of vitiligo by many groups [7]. The histological analysis of the perilesional margin surrounding the patients’ de-pigmented skin revealed infiltrating of activated T cells and other lymphocytes [8]. Further studies confirmed that these surrounding T cells were skin-homing, and were apparently cytotoxic to nearby melanocytes [9], [10]. Melanocyte-specific antibodies were often observed in vitiligo patients’ serum [11] and in melanocyte-specific skin-homing cytotoxic T lymphocytes (CTLs) [12]. In the perilesional vitiligo skin, these CTLs are correlated with the clinical presentations of vitiligo. CTLs were shown to induce apoptosis of melanocytes in the non-lesional skin [13]. These studies confirmed the involvement of autoimmune T cells in the pathological mechanisms of vitiligo.

Clinically, transplantation of autologous melanocytes therapy is proven to be the most effective therapy option for the vitiligo patients [14], [15], [16]. Groups including ours have treated vitiligo patients using this method with the cure rate above 53% [14], [15]. Studies showed that the number of cytotoxic CD8+ T cells was higher in active disease than in stable disease [17]. Recent study suggested that cytotoxic CD8+ T cells in vitiligo perilesions may dictate the fate of transplantation [17], and strategies against CD8+ T cell activation might be beneficial for patients undergoing melanocyte transplantation. Clinical evidence to support this notion is, however, still missing.

Friedenstein and colleagues first characterized mesenchymal stem cells (MSCs) from adult bone marrow, which were an adherent, fibroblast-like cell population [18]. Since then, different groups have also isolated MSCs from other tissues including adipose, cord blood, and fetal liver, blood, fetal pancreas, and lung [19], [20], [21]. MSCs have capacities to self-renew and differentiate into various cells with mesenchymal origins [18]. Recent studies have been focusing on the immune-regulatory potential of MSCs. It appeared that MSCs-mediated immunosuppressive activity was major histocompatibility complex (MHC) independent, and CD4+ and CD8+ T cells were equally susceptible to MSCs [22], [23]. MSCs could inhibit T cell proliferation and induce T cell apoptosis [24], [25]. Further, studies revealed that MSCs inhibited T cell activation and caused T cell unresponsive [24], [25]. The immunomodulatory effects of MSCs may be used to repair tissue damages caused by the immune system, and to prevent rejection of organ transplants [22], [23]. However, the potential effects of MSCs on skin-homing T cell activities are not extensively studied.

Dermal stem cells were originally isolated from the dermis of juvenile and adult mice by Toma et al., [26], afterwards, same group indentified such a cell population in human skin [27]. Georg Bartsch firstly indentified and characterized dermal mesenchymal stem cells (DMSCs) [28]. DMSCs had multi-lineage differentiation potential into adipocytes, osteocytes and chondrocytes [28]. The surface antigenic profile of DMSCs was positive for CD90 but differs in regard to negativity for CD34 [28]. To our knowledge, the potential immunomodulatory effects of DMSCs on skin-homing T cells are not studied. In the current study, we were set to investigate the factors determining the efficiency of autologous melanocyte transplantation of vitiligo patients by focusing on perilesional skin homing CD8+ T lymphocytes, and studied the potential effects of dermal mesenchymal stem cells (DMSCs) on CD8+ T cell activities *in vitro*.

## Materials and Methods

### Patients and Ethics

Twenty-three vitiligo patents undergoing autologous melanocytes transplantation at the Third People’s Hospital of Hangzhou were included in this study. For all 23 patients, the vitiligo lesions were stable for at least one year with no new lesions developed. The study was approved by the institutional review board of Third People’s Hospital of Hangzhou, and written informed consent was obtained from all patients. All clinical investigation was conducted according to the principles expressed in the Declaration of Helsinki. The gender, period of stabilization, type of vitiligo, location and size of vitiligo area in these patients were included in Table 1.

**Table 1:** Demographic and clinical data of vitiligo patients undergoing transplantation.

	Results of transplantation			
	Excellent	Good	Fair	Poor
Number of case	12	6	3	2
Age	31.1±3.4	28.3±4.1	30.5±5.8	33.2±6.1
Gender (male/female)	5/7	4/2	1/2	2/0
Type of vitiligo (localized/generalized)	12/0	6/0	3/0	2/0
Period of stability (month)	25.7±22.2	23.2±12.3	22.4±20.1	25.1±18.2
Size of lesion (cm^2^)	28.9±6.7	15.8±8.6	26.7±14.2	30.1±22.3
Cell density (melanocytes/mm^2^) for graft	778±176	760±139	728±129	745±132

### Autologous Melanocytes Transplantation Procedure

The patients’ autologous melanocytes were isolated and cultured as described previously [14], [15]. After the number of melanocytes met the requirement for transplantation, the melanocytes were dissociated and re-suspended in F12 medium, and were immediately transferred to the operating room for transplantation. The recipient areas treated with lidocaine cream were cleaned with 70% alcohol, and the epidermis was removed with ultra pulse CO_2_ laser. The melanocytes suspension was applied to the laser-denuded area at a density of 600–1000 cells/mm^2^. Afterwards, the transplant area was covered with Vaseline gauze, followed by gauze soaked with F12 medium and finally secured with gauze and surgical tape. A 3-mm punch biopsy was taken from the margin of the patch (perilesional area) on the day of transplantation for immunohistochemistry study. Meanwhile, the fluid under epidermis of recipient area (vitiligo area) was collected by suction blister before removing the epidermis completely with ultra pulse CO_2_ laser, cytokines levels in the fluid were analyzed.

### Transplantation Therapy Efficiency Evaluation

Re-pigmentation of receipt area was evaluated 10 days, 1, 2, 3 and 6 months after the transplantation. The re-pigmented area was measured. Different category of treatment responses was defined according to the extent of re-pigmentation: excellent, 90% or more re-pigmentation rate; good, 50–89% re-pigmentation rate; fair, 20–49% re-pigmentation rate, and poor, less than 20% re-pigmentation rate.

### Protein Array Analysis of Chemokines and Cytokines

The expression profile of 79 different cytokines in culture supernatants of skin-homing CD8 T cells, or in fluid under epidermis of patients vitiligo area were assessed semi-quantitatively using RayBio™ Human Cytokine Array V (RayBiotech, Norcross, GA) according the manufacturer’s protocol.

### Immunohistochemistry (IHC)

The perilesional skin tissues were fixed by 10% neutral buffered formalin and were embedded in paraffin and affixed onto the slide of 1–5 mm thickness. The slides were de-paraffinized and placed in microwave oven and boiled for 20 min followed by cooling for 20 min in room temperature (RT). Endogenous peroxidase was blocked by incubation with 0.25% hydrogen peroxide (H_2_O_2_, Sigma, St. Louis, MO), and 0.001% sodium-azide in TBS for 20 min at RT. Subsequently, the sections were incubated with 10% normal goat serum (Gibco, Shanghai, China) in TBS for 15 min at RT. The mouse anti-human CD8 mAb (1∶100, BD Biosciences, San Jose,CA) were added to slides for 60 min at RT in TBS with 1% bovine serum albumin (BSA). The bound antibody was then detected by streptavidin-HRP (1∶400, Dako Corporation, Carpinteria, CA) and antibody reactivity was detected by incubation with DAB substrate, according to the manufacturer’s instructions. The sections were counterstained with hematoxylin (Sigma) for 1 min at room temperature, and cover slips were mounted using Kaiser’s glycerol gelatin (Sigma). The number of CD8 positive cells and lymphocytes were recorded in 10 high power fields, the percentage of CD8 positive cells was calculated.

### Primary Culture of Human DMSCs

Human DMSCs were obtained from dermis of human foreskin. The epidermis was manually removed from tissue piece and incubated in dispase (Gibco) overnight at 4°C, then the dermis was cut into 1-mm^3^ pieces and incubated in collagenase II (Gibco) for 1 h at 37°C, and 10% fetal bovine serum (FBS, Gibco) was added to neutralize the enzymes. The skin tissue was manually dissociated by pipetting repeatedly and the cell suspension was centrifuged at 1000 rpm for 5 min. The supernatant was removed and the pellet was re-suspended in 10 ml DMEM (Gibco) containing 10% FBS and 1% penicillin/streptomycin (Gibco). Afterwards, the cell suspension was transferred to a 25-cm^2^ non-tissue culture flask. About 2 hrs later, when a small group of cells adhered to the flask, the medium with un-adhered cell was removed. The expression levels of CD11b, CD90, CD44, CD73, CD105, CD45, CD19, and CD34 in passage 3 DMSCs were tested using flow cytometry as described [29].

### Primary Culture of Human Skin Homing CD8+ T Cells

As previously described [29], skin homing CD8+ T cells were obtained from biopsies of Halo nevus [30]. The biopisies (2 mm) were cultured in 24-well plates in 1 ml RPMI 1640 (Gibco) supplemented with 10% FBS, 0.1% penicillin-streptomycin, 2 mM L-glutamine (Gibco), 2 ng/mL IL-2 (PeproTech), 5 ng/mL IL-15 (PeproTech). In addition, 1.25 µl/mL anti-CD3/CD28 mAb-coated T-cell expander beads (Miltenyi Biotec) were added to promote T-cell outgrowth. CD8+ T cells were separated with human CD8 T cell isolation kit according to manufacturer’s protocol (Miltenyi Biotec, Germany).

### Assay of *in vitro* Human Skin CD8+ T cells Proliferation when Co-cultured with DMSCs

As reported [29], Skin CD8+ T cells were collected and re-suspended in pre-warmed PBS at a final concentration of 1×10^6^ cells/ml. To this was added 1 µl of CFSE (carboxyfluorescein diacetate, succinimidyl ester, 5 µM) dye at 37°C for 10 min. The staining was then quenched by the addition of 5 volumes of ice-cold culture media. Cells were then pelletted, re-suspended and washed in fresh media for three times. Stained T cells were then cultured with DMSCs (with ratio 1∶1) in appropriate conditions. Afterwards, cells were harvested, and CFSE fluorescence was analyzed using a flow cytometer with 488 nm excitation and emission filters.

### Assay of *in vitro* Human Skin CD8+ T cells Apoptosis when Co-cultured with DMSCs

As described, perilesional CD8+ T cells were co-cultured with DMSC at the ratio of 1∶1 for 3 days, cells were detached and incubated in 500 µl binding buffer, 5 µl annexinV-FITC and propidium iodide (PI, Invitrogen, Shanghai, China) of each at room temperature for 5 min in the dark. Cell apoptosis histograms were generated after analysis of PI-stained cells by fluorescence-activated cell sorting (FACS) with a Becton-Dickinson FACScan.

### Statistical Analysis

For experiments in this study, individual culture dishes or wells were analyzed separately (no pooling of samples was used). In each experiment, a minimum of three wells/dishes was used and similar results were obtained. Each experiment was repeated a minimum of three times, the mean value of the repetitions was calculated and this value was used in the statistical analysis. Data are presented as mean±SD. The differences were determined by one-way ANOVA in appropriate experiments followed by Newman–Keuls post hoc test. A probability value of *p*<0.05 was taken to be statistically significant.

## Results

### Efficacy of Transplantation

The efficiency of transplantation therapy was evaluated by re-pigmentation percentage of the vitiligo lesion 12 months after procedure in all 23 patients. Results in Table 1 demonstrated that 12 (52.17%) patients had an excellent response with more than 90% or more re-pigmentation rate, 6 (26.09%) patients had a good response (50–89% re-pigmentation rate), 3 (13.04%) patients had a fair response (20–49% re-pigmentation rate) and 2 (8.70%) patients received poor re-pigmentation (less than 20% re-pigmentation rate). Other demographic and clinical data was also included in Table 1. No scars or other complications were observed in the donor sites or recipient sites (Data not shown). Koebner phenomenon was not observed at donor sites in all patients (Data not shown).

### Perilesional CD8+ T cell Infiltration is Associated with the Re-pigmentation Efficiency of Patients with Autologous Melanocytes Transplantation

**Figure 1:**
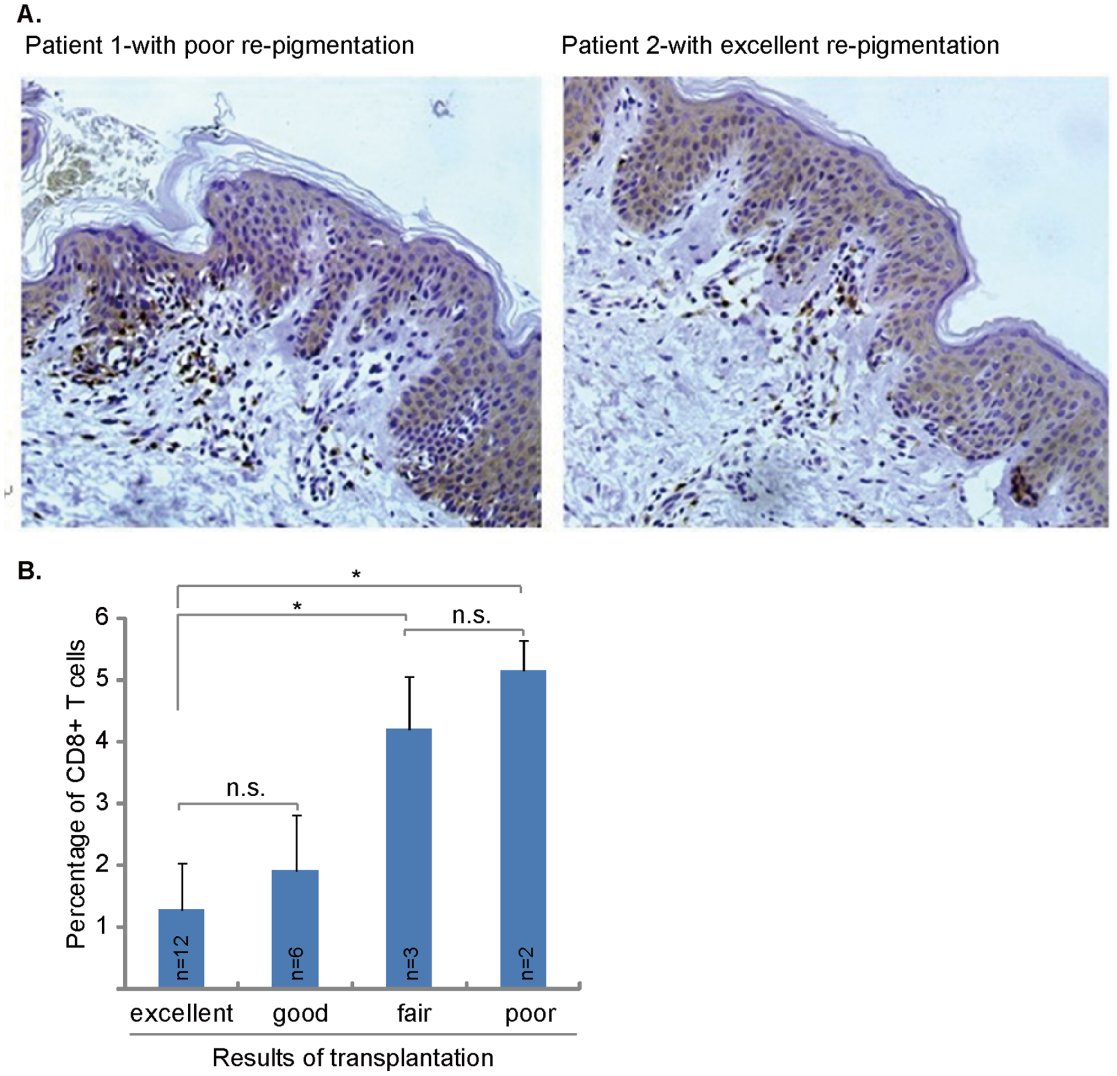
Perilesional CD8+ T cell infiltration is associated with the re-pigmentation efficiency of patients with autologous melanocytes transplantation. Presence of CD8+ T cells in the perilesional skin of vitiligo lesions. Immunohistochemistry analysis of the perilesional skin of vitiligo patients revealed CD8^+^ T-cell infiltrations (DAB staining) (A). Patient 1 obtained 30% re-pigmentation after transplantation, patient 2 obtained 85% re-pigmentation after transplantation. Photos show the anti-CD8 mAb (brown) staining of two representative patients. Statistical analysis shows that the difference of CD8+ T cell infiltrating mainly exist between patients with fair/poor re-pigmentation and patients with excellent re-pigmentation (B). *p<0.05. n.s.: no statistical difference. Immunohistochemistry staining, original magnification ×200 (A and B).

Significant CD8+ T cells infiltrating was observed in the perilesional vitiligo area of all patients, even these vitiligo areas were stable for at least 12 months (Figure 1A). Importantly, the patient’s prognosis, or the re-pigmentation rate of vitiligo area after transplantation, was closely associated with number of perilesional skin-homing CD8+ T cells. The patients with high number of perilesional CD8+ T cells were associated with poor re-pigmentation rate or poor prognosis (Figure 1A and B). On the other hand, we observed a significant lesser number of CD8+ T cells infiltrating in patients with excellent or good re-pigmentation responses (Figure 1A and B). The mean percentage of infiltrated CD8+ T cells in patients with fair or poor re-pigmentation responses was significantly higher than that in patients with excellent or good re-pigmentation responses (Figure 1B). These results suggest that CD8+ T cell perilesional infiltration is associated with the re-pigmentation efficiency of patients with transplantation.

### Cytokines and Chemokines Expression Levels in Epidermis Fluid of Vitiligo Area are Associated with the Re-pigmentation Efficiency of Patients with Autologous Melanocytes Transplantation

**Figure 2:**
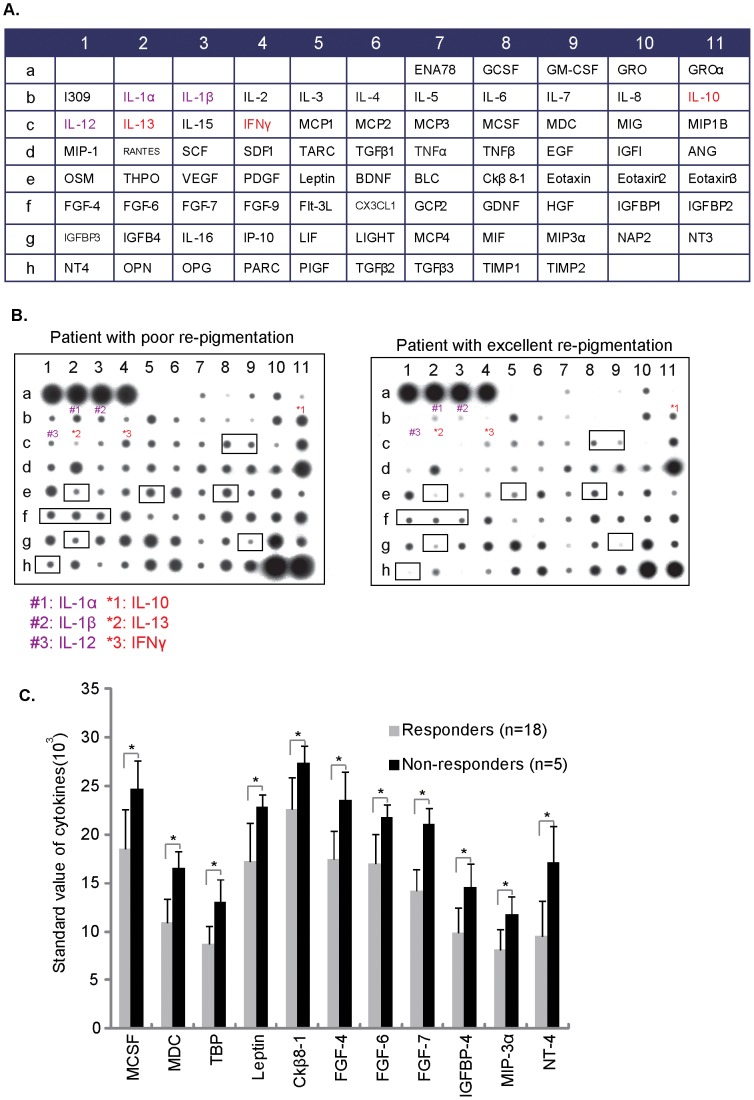
Cytokines and chemokines expression levels in epidermis fluid of vitiligo area are associated with the re-pigmentation efficiency of patients with autologous melanocytes transplantation. The results of 79 total cytokines/chemokines (A) array for the fluid under epidermis of recipient area in patients with poor or excellent re-pigmentation (A–B). 11 cytokines between non-responder group and responder group was statistically significant (C). *p<0.05. Abbreviations: THPO: Thrombopoietin, Flt-3 L: Flt-3 Ligand, OSM: Oncostatin M, OPG: osteoprotegerin, OPN: steopontin, ANG: angiogenin, MCP: monocyte chemotactic protein and MDC: macrophage-derived chemokine.

A total of 79 cytokines and chemokines (Figure 2A) in the fluid under epidermis of recipient area were assessed using RayBio™ Human Cytokine Array V as described in two groups: patients with excellent re-pigmentation (responders, n = 12) and patients with poor or fair re-pigmentation (non-responders, n = 5). Results in Figure 2B showed that the levels of pro-inflammatory cytokines (such as IL-1α, IL-1β and IL-12 in “purple”) and CD8+ T cell associated cytokines (such as IL-10, IL-13 and IFNγ, in “red”) [31], [32], [33] were much higher in epidermis fluid of patients with poor re-pigmentation (Figure 2B). Further, a total of 11 other cytokines/chemokines levels were significant higher in non-responders group (Figure 2C). These results together suggest that cytokines and chemokines expression levels in epidermis fluid of vitiligo area are associated with the re-pigmentation efficiency of transplantation patients.

### Human DMSCs Isolation and Characterization

**Figure 3:**
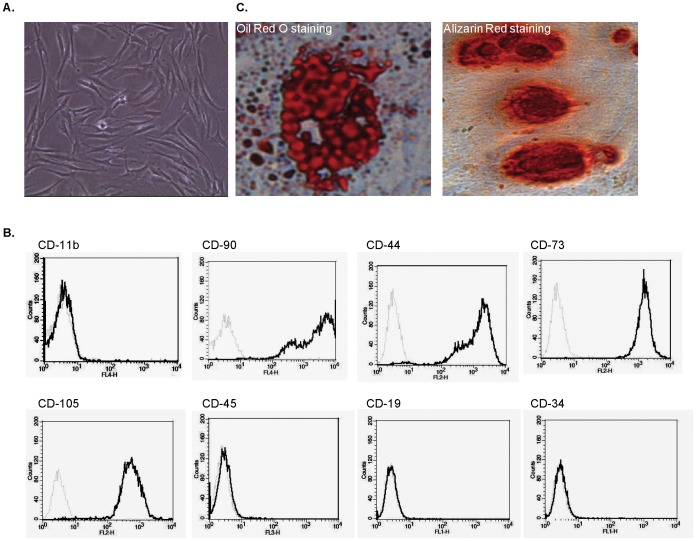
Human DMSCs isolation and characterization. DMSCs showed typical adherent fibroblast-like morphology (A), cells were uniformly positive for CD44, CD73, CD90, CD105 and negative for CD11b, CD19, CD34, CD45 (B) and cultured DMSCs (passage 3) differentiated into adipocytes and osteoblasts when cultured in appropriate differentiation culture conditions as mentioned (C). Each experiment was repeated three times and similar results were obtained.

DMSCs were successfully isolated from the dermis of foreskin and expanded *in vitro*. DMSCs proliferated well in culture under passage 15 and showed typical adherent fibroblast-like morphology (Figure 3A). The phenotype profile of DMSCs was confirmed by flow cytometry, as these cells were uniformly positive for CD44, CD73, CD90, CD105 and negative for CD11b, CD19, CD34, CD45 (Figure 3B) [34]. DMSCs in passage 3 were tested for their ability to differentiate into adipocytes and osteoblasts. Adipocytes differentiation was induced by 1 µM of dexamethasone, 10 µg/ml of insulin, 0.5 mM of methylisobutylxanthine and 100 µM of indomethacin. Differentiated adipocytes were stained by oil red (Figure 3C, left). Osteoblasts differentiation was induced by 100 nM of dexamethasone, 10 mM of β-glycerophosphate, and 0.05 mM of ascorbic acid. Differentiated osteoblasts are stained with alizarin red (Figure 3C, right).

### Human DMSCs Suppress Skin Homing CD8+ T cell Proliferation and Induce Apoptosis

**Figure 4:**
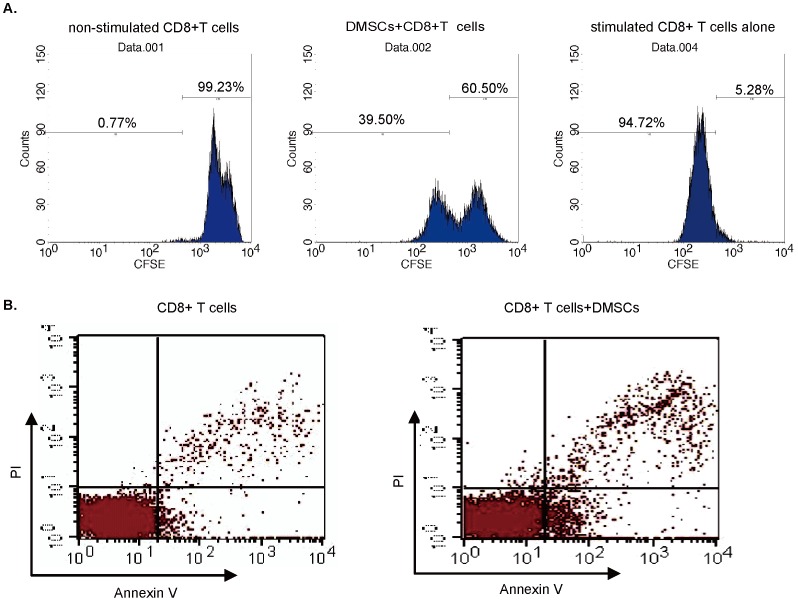
Human DMSCs suppress skin homing CD8+ T cells proliferation and induce apoptosis. CD8+ T cell proliferation was assayed using CFSE dilution with FACS analysis (A), results showed that DMSCs resulted in significant inhibition of CD8+ T cell proliferation (A). The apoptosis of CD8+ T cells co-cultured with or without DMSCs was analyzed by flow cytometry detecting PI stained cells (B). Each experiment was repeated three times and similar results were obtained.

The T cells isolated from Halo nevus were uniform for CD8 positive (data not shown), similar to our previous publication [29]. Results in Figure 4A demonstrated that skin homing CD8+ T cells cell proliferation was significantly inhibited when co-culture with DMSCs at 1∶1 ratio (CD8+ T cells proliferation was analyzed by CFSE FACS as described in material and methods), note that the percentage of proliferative CD8+ T cells dropped from 94.72% to 39.50% (*p*<0.05) after DMSCs co-culture (Figure 4A). Further, adding DMSCs also induced CD8+ T cell apoptosis. In control group where no DMSCs were added, the apoptosis percentage of CD8+ T cells was 3.2±0.6%. The percentage jumped to 9.6±1.5% (*p*<0.05) when co-cultured with DMSCs (Figure 4B). These results together showed that DMSCs suppress skin homing CD8+ T cell proliferation and induce apoptosis in the co-culture system.

### Human DMSCs Inhibit Skin Homing CD8+ T cell Cytokines and Chemokines Production

**Figure 5:**
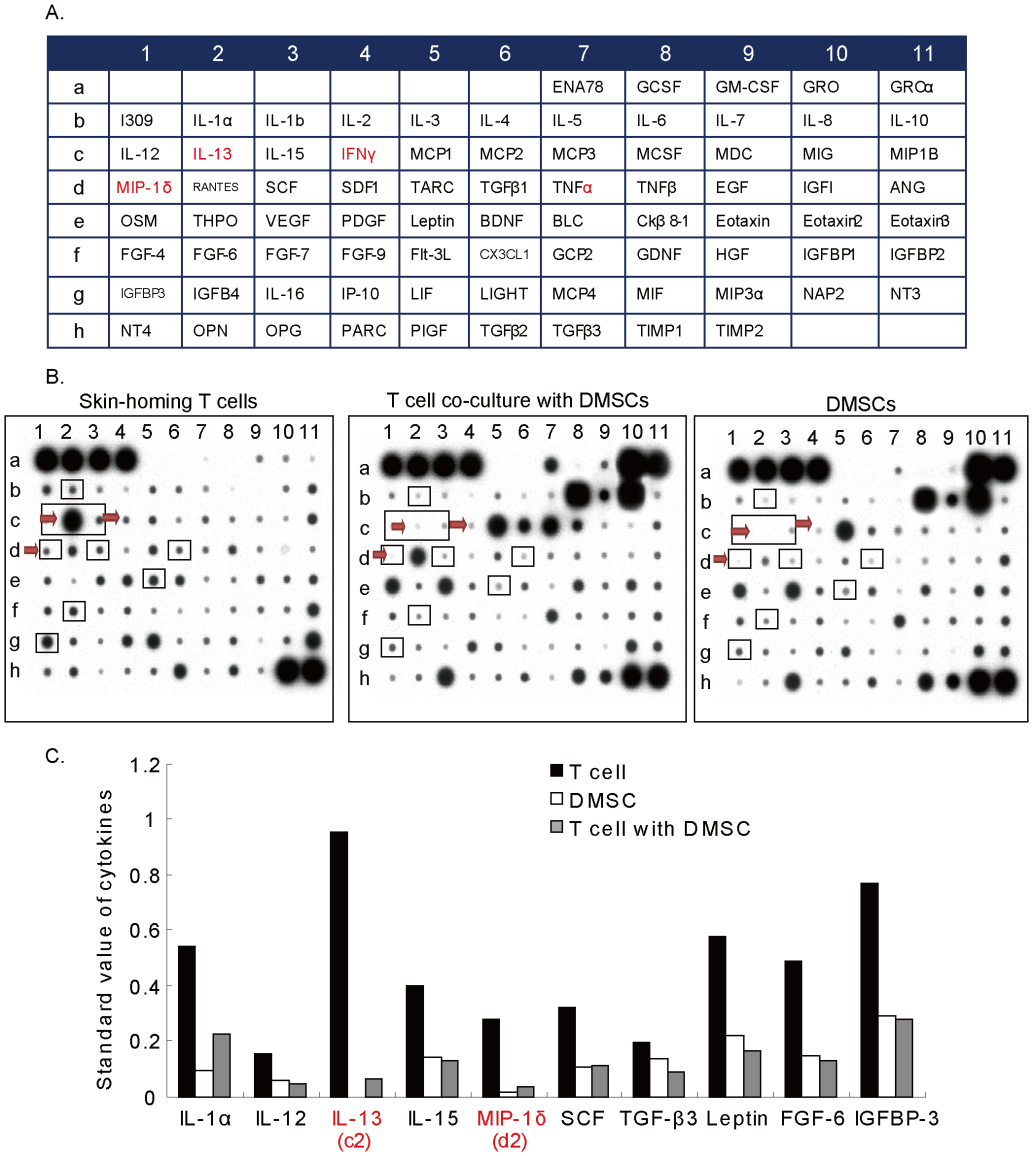
Human DMSCs inhibit skin homing CD8+ T cell cytokines and chemokines production. The cytokines profiles of CD8+ T cells modulated by DMSCs. The concentration of 79 cytokines (A) in supernatants from DMSCs/CD8+ T cells co-culture, DMSCs monoculture and CD8+ T cells monoculture was detected (B). The ten cytokines mostly affected by co-culture system were shown in C.

We then tested cytokines profile in skin-homing CD8+ T cells and studied the effects of DMSCs in the same co-culture system. The concentrations of a total 79 cytokines in supernatants from human skin homing CD8+ T cells with or without DMSCs co-culture were measured (Figure 5A). Results in Figure 5B and C showed that DMSCs co-culture significantly inhibited the production of a largely number of cytokines and chemokines including IL-13 and MCP-1 (red arrow) (Figure 5B). We also listed 10 cytokines/chemokines that were mostly affected by DMSCs co-culture (Figure 5B and C). Results indicate that human DMSCs significantly inhibit cytokines and chemokines production by skin homing CD8+ T cell.

## Discussion

Different groups have confirmed the presence of CD8+ cytotoxic T cells clustering around the de-pigmented vitiligo skin lesion [12], which is strongly associated with the disease development [13], [35], [36]. Furthermore, the patients with a stronger CD8+ T cell reactions often need a strong UVB irradiation before the first re-pigmentation occurs [13], [35], [36]. Adoptive transfer of melanocyte antigen-specific T cells to vitiligo lesions is now known as the major characteristic of vitiligo [16], [35]. These perilesional T cells are able to kill local melanocytes directly within their physiological microenvironment [16], [35].

In the current study, we observed significant CD8+ T cells infiltrating in the perilesional vitiligo area of all patients, even these vitiligo areas were considered stable for at least 12 months. Importantly, the efficiency of the transplantation, or the re-pigmentation of vitiligo area after transplantation, was closely associated with skin-homing CD8+ T cell activities. The patients with high number of perilesional CD8+ T cells or high levels of Th cytokines/chemokines were associated with poor re-pigmentation efficiency (poor prognosis). Thus, we suggest that the extent of infiltrating CD8+ T cell in vitiligo perilesions may predict the outcomes of transplantation. Based on our preliminary results, we recommend that the level of CD8+ T cell infiltration in vitiligo perilesions may need to be evaluated before melanocytes graft, and the efficacy of transplantation may be improved when accompanied by strategies against local CD8+ T cells. Large sample study is, however, needed to further support our hypothesis.

Meanwhile, we observed the concentration of at least 11 other cytokines was significantly higher in the fluid under lesional area of patients with poor transplantation prognosis, which indicates that these cytokines may also be linked to transplantation efficiency to certain extent (Figure 2C). However, the relationship between these cytokines and vitiligo development/prognosis needs further investigations. For example, the level of leptin was significant higher in non-responders, while leptin is known to play a important role in inflammation and autoimmunity [37], and increased secretion of leptin is associated with chronic inflammatory [37]. Interestingly, we also observed a significant high level of leptin in supernatant of skin homing CD8+ T cells (Figure 5), and DMSCs significantly inhibited leptin secretion (Figure 5).

In addition to their differentiative properties, mesenchymal stem cells (MSCs) have shown significant immunosuppressive and anti-inflammatory abilities [22], [25], [38], [39]. We here successfully isolated and characterized dermal mesenchymal stem cells (DMSCs) from the adult human foreskin. In conditional medium, these cells showed the abilities to differentiate into adipocytes or osteocytes (Figure 3). FACS analysis results showed that these cells were positive for CD44, CD73, CD90, CD105, but negative for CD11b, CD19, CD34, CD45 to indicate that these cells are a population of functional MSCs. MSCs possess an arsenal of immunosuppressive mechanisms, which can be deployed in the modulation of inflammation. Studies have shown that mouse MSCs can also chemoattract T lymphocytes through the secretion of CXCL9, CXCL10 and CCL2 [40], [41]. Once MSCs have attracted effector T cells, this provides a platform for MSCs contact with effector T cells and facilitates the direct immunomodulation of the T cells via production NO by MSCs [41] or Fas/FasL –induced apoptosis [40]. Recent studies have shown that MSCs may directly induce apoptosis of activated T cells [25], [42]. It was suggested that this apoptosis might be associated with the conversion of tryptophan into kynurenine by indoleamine 2,3-dioxygenase expressed by MSCs [25]. Further, MSCs-derived NO was shown to induce apoptosis of T cells through suppression of STAT-5 phosphorylation [43]. In the co-culture system, our data showed that DMSCs significantly inhibited skin homing CD8+ T proliferation and induced those cells apoptosis. Further, DMSCs significantly inhibited secretion of multiple cytokines/chemokines by skin homing CD8+ T cells. These cytokines including pro-inflammatory cytokines IL-12, TNFα and IL-1α [44], immuno-regulatory cytokines TGF-β and more importantly, Th associated cytokines IL-13, IFN-γ and MIP-1 (Figure 5, red arrow). These data confirm that DMSCs induces significant immunosuppressive abilities against skin homing CD8+ T lymphocytes and may help improve the efficacy of melanocytes transplantation.

### Conclusion

In conclusion, we demonstrated significant CD8+ T cells infiltrating in the perilesional area of vitiligo patients undergoing melanocyte transplantation. The efficiency of vitiligo patients’ autologous melanocytes transplantation is closely associated with skin-homing CD8+ T cell activities. DMSCs inhibit CD8+ T cells proliferation, induce them apoptosis and regulate their cytokines/chemokines production. Our results suggest that vitiligo patients’ autologous melanocytes transplantation efficiency may be predicted by perilesional skin-homing CD8+ T cell activities, and the immuneregulatory DMSCs might be used as auxiliary agent to improve the efficacy.
